# Acquired Hemophilia A in an Elderly Patient Receiving Clopidogrel: Drug-Induced or Age-Associated Autoimmunity?

**DOI:** 10.7759/cureus.113716

**Published:** 2026-07-31

**Authors:** Akhil Mohanachandran Pushpaleela, Rahil Omar Tai Valappil, Ayshath Hansiya, Siobhan Lewis

**Affiliations:** 1 Acute Medicine, Cardiff and Vale University Health Board, Cardiff, GBR; 2 General Medicine, Cardiff and Vale University Health Board, Cardiff, GBR; 3 Geriatrics, Cardiff and Vale University Health Board, Cardiff, GBR

**Keywords:** acquired coagulation disorder, acquired haemophilia a, case report, clopidogrel, elderly patient, factor viii inhibitor, haemorrhagic complications, spontaneous bleeding

## Abstract

Acquired haemophilia A (AHA) is a rare but potentially life-threatening autoimmune bleeding disorder caused by autoantibodies against factor VIII. Its presentation is often non-specific and can mimic more common causes of bleeding, leading to delayed diagnosis, particularly in elderly patients. We report the case of a 93-year-old man with multiple comorbidities who presented with spontaneous, extensive bruising and haemorrhagic complications. Laboratory evaluation demonstrated findings consistent with AHA, including isolated prolongation of the activated partial thromboplastin time (aPTT), reduced factor VIII activity, and the presence of factor VIII inhibitors. The patient received haematology-directed treatment; however, his clinical course was complicated, and he ultimately died. This case highlights the importance of maintaining a high index of suspicion for AHA in elderly patients presenting with unexplained bleeding and emphasises the need for prompt diagnosis and early multidisciplinary management.

## Introduction

Acquired hemophilia A (AHA) is a rare autoimmune bleeding disorder with an estimated incidence of 1-2 cases per million annually, although its incidence increases significantly with advancing age [1,2]. It is caused by the development of autoantibodies directed against coagulation factor VIII, resulting in impaired hemostasis and spontaneous bleeding, most commonly involving the skin and soft tissues [3]. Unlike congenital hemophilia, AHA typically occurs in individuals with no personal or family history of bleeding disorders. Approximately half of AHA cases are idiopathic, while the remainder are associated with underlying conditions such as malignancy, autoimmune disorders, and medications; AHA is also more common in older adults, who frequently have multiple comorbidities [4].

Because of its rarity and often non-specific presentation, early diagnosis remains challenging and requires a high index of suspicion. An isolated prolonged activated partial thromboplastin time (aPTT), together with measurement of factor VIII activity and factor VIII inhibitor testing, plays a key role in establishing the diagnosis. Mixing studies may help differentiate a factor deficiency from an inhibitor but cannot independently establish or exclude AHA. Prompt recognition is essential, as diagnostic delays contribute to increased morbidity and mortality [5]. We present a case of AHA in an elderly patient receiving clopidogrel, highlighting the diagnostic challenges, potential drug association, and importance of early recognition and multidisciplinary management.

## Case presentation

A 93-year-old retired bricklayer residing in a care home was admitted to the hospital with multiple symptoms, including increasing fatigue, weakness, recurrent falls, extensive bruising, shortness of breath, and progressive immobility. His medical history included mixed dementia, ischemic heart disease with previous coronary artery bypass grafting, heart failure, hypertension, and a permanent pacemaker. His regular medications included clopidogrel, prescribed for secondary prevention following coronary artery bypass grafting, along with ramipril, lansoprazole, atorvastatin, and donepezil.

On examination, he was agitated, disoriented, and hypoxic, with an oxygen saturation of 85% on room air. Extensive bruising was noted over the anterior neck, base of the tongue, and forearms, later progressing to involve the legs, feet, spine, and flanks. Airway compromise developed secondary to mucosal swelling, and speech and swallowing assessments demonstrated severe oropharyngeal dysphagia. Cardiovascular and abdominal examinations were otherwise unremarkable.

**Figure 1:**
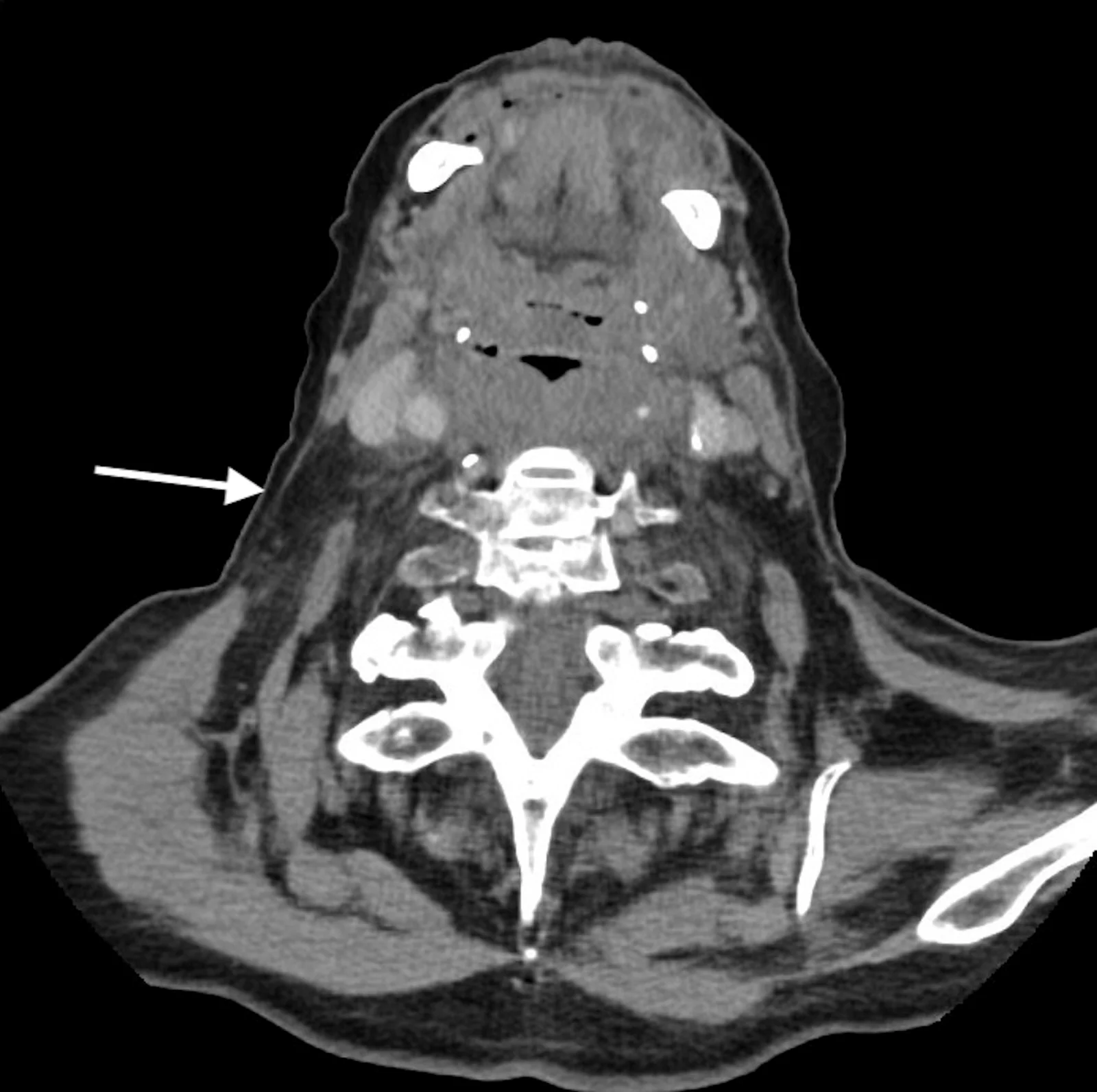
CT image of the neck demonstrating a hematoma within the right sternocleidomastoid muscle.

**Figure 2:**
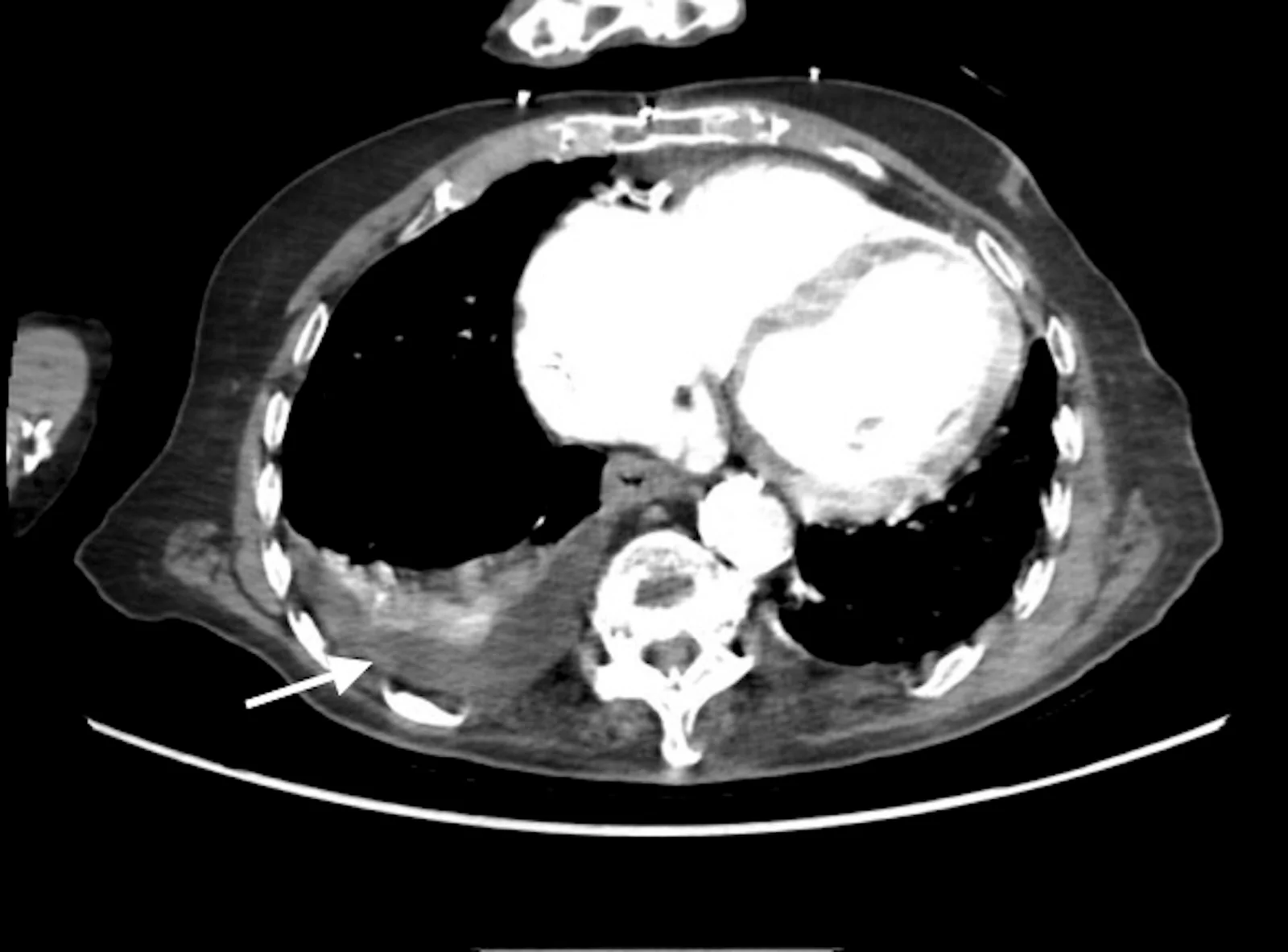
CT image of the chest demonstrating a right pleural effusion with a possible hemothorax.

Initial laboratory investigations revealed an isolated prolonged aPTT, without thrombocytopenia or other findings suggestive of disseminated intravascular coagulation (DIC). A mixing study demonstrated persistent prolongation of the aPTT without correction, suggesting the presence of a coagulation factor inhibitor. CT of the neck demonstrated extensive bilateral anterior neck soft-tissue swelling and fat stranding, with asymmetric enlargement of the right sternocleidomastoid muscle, consistent with an intramuscular hematoma (Figure 1). Diffuse mucosal edema involving the oropharynx and supraglottis resulted in significant narrowing of the oropharyngeal and laryngeal airway. No contrast extravasation or pooling was identified to suggest active hemorrhage, and the major neck vessels opacified normally. CT imaging of the chest demonstrated a small right pleural effusion; a small hemothorax could not be entirely excluded (Figure 2). No traumatic solid-organ injury, acute bony injury, or radiological source of active bleeding was identified. Following hematology consultation, coagulation factor analysis demonstrated undetectable factor VIII activity, while factor IX testing was pending. These findings were consistent with AHA.

The patient was commenced on immunosuppressive therapy with corticosteroids and mycophenolate mofetil to reduce inhibitor burden. Clopidogrel, which has been reported in association with AHA, was discontinued because of life-threatening active bleeding and the possibility of drug-associated AHA. Hemostatic treatment with recombinant porcine factor VIII (OBIZUR) was initiated at 6000 units and subsequently escalated to 10,000 units due to inadequate clinical response. Additional supportive management included tranexamic acid and RBC transfusions.

Despite treatment, factor VIII levels remained persistently low. Further testing identified the presence of a porcine factor VIII inhibitor, suggesting reduced responsiveness to OBIZUR. Consequently, treatment was escalated to include factor eight inhibitor bypassing activity (FEIBA) and rituximab. However, the patient’s clinical condition continued to deteriorate, with progressive bruising and uncontrolled bleeding. Despite intensive medical management, hemostasis could not be achieved, and the patient subsequently died.

## Discussion

This case underscores the clinical challenges involved in diagnosing and managing AHA. Although the diagnosis was established within hours of the patient’s presentation to the ED, the complex nature of AHA made subsequent management extremely challenging. Recognition of this condition remains difficult because of its rarity, limited clinician familiarity, and potential for confusion with more common causes of bleeding [6]. Spontaneous bruising in the absence of trauma, particularly when associated with an isolated prolonged aPTT, should prompt consideration of AHA [7].

Clopidogrel, which the patient was receiving, has been associated with drug-induced AHA in several case reports, although establishing a definite causal relationship remains challenging. Previous pharmacovigilance analyses using the WHO VigiBase database have also suggested a possible association between clopidogrel exposure and the development of AHA.

Management of AHA is generally centered on two objectives: achieving hemostatic control and eradicating the inhibitor [8]. First-line immunosuppressive treatment typically consists of corticosteroids alone or in combination with cytotoxic agents or rituximab [9]. For bleeding control, bypassing agents such as activated prothrombin complex concentrate (aPCC; FEIBA) or recombinant activated factor VII (rFVIIa) are commonly used for clinically relevant bleeding [10]. In our patient, despite treatment escalation involving corticosteroids, mycophenolate mofetil, recombinant porcine factor VIII (OBIZUR), FEIBA, and rituximab, bleeding remained uncontrolled. Elderly patients with multiple comorbidities may demonstrate a limited treatment response, and their overall prognosis remains guarded [11].

Although bypassing agents such as FEIBA and recombinant activated factor VII are effective in controlling bleeding, they are associated with a potential risk of thromboembolic complications. This risk may be particularly relevant in elderly patients with pre-existing cardiovascular disease, requiring individualized treatment decisions and careful multidisciplinary monitoring to balance hemostatic efficacy against thrombotic risk.

Although its role in AHA continues to evolve, emerging evidence suggests that emicizumab may offer an effective alternative for the management of acute bleeding episodes, with fewer thrombotic complications compared with bypassing agents and reduced duration of hospitalisation [9]. Although its role in AHA continues to evolve, emerging evidence suggests that emicizumab may offer an effective alternative for the management of acute bleeding episodes, with fewer thrombotic complications compared with bypassing agents. While emicizumab was not used in our patient, its evolving role may influence future treatment strategies, particularly in elderly patients with refractory disease.

## Conclusions

AHA should be considered in elderly patients presenting with spontaneous, unexplained bleeding and an isolated prolonged aPTT, particularly in the absence of a known bleeding disorder or when patients are receiving potentially associated medications such as clopidogrel. Early recognition and prompt initiation of immunosuppressive and hemostatic therapy may improve clinical outcomes. This case reinforces the importance of maintaining clinician awareness of rare but serious conditions such as AHA in geriatric populations. Although emicizumab was not used in this case, emerging evidence suggests that it may represent a promising adjunctive or alternative therapeutic option, particularly for refractory disease or in selected prophylactic settings.

## References

[1] Collins PW, Hirsch S, Baglin TP (2007). Acquired hemophilia A in the United Kingdom: a 2-year national surveillance study by the United Kingdom Haemophilia Centre Doctors' Organisation. Blood.

[2] Knoebl P, Marco P, Baudo F (2012). Demographic and clinical data in acquired hemophilia A: results from the European Acquired Haemophilia Registry (EACH2). J Thromb Haemost.

[3] Franchini M, Lippi G (2008). Acquired factor VIII inhibitors. Blood.

[4] Kruse-Jarres R, Kempton CL, Baudo F (2017). Acquired hemophilia A: updated review of evidence and treatment guidance. Am J Hematol.

[5] Shander A, Walsh CE, Cromwell C (2011). Acquired hemophilia: a rare but life-threatening potential cause of bleeding in the intensive care unit. Intensive Care Med.

[6] Collins PW (2011). Management of acquired haemophilia A. J Thromb Haemost.

[7] Baudo F, Collins P, Huth-Kühne A (2012). Management of bleeding in acquired hemophilia A: results from the European Acquired Haemophilia (EACH2) Registry. Blood.

[8] Huth-Kühne A, Baudo F, Collins P (2009). International recommendations on the diagnosis and treatment of patients with acquired hemophilia A. Haematologica.

[9] Launois A, Martin-Toutain I, Devaux F (2024). [Acquired hemophilia A and emicizumab for the treatment of bleeding: two case report and a literature review]. Ann Biol Clin (Paris).

[10] Alcedo Andrade PE, Mannucci PM, Kessler CM (2024). Emicizumab: the hemophilia A game-changer. Haematologica.

[11] Konstantinov K, Dolladille C, Gillet B, Alexandre J, Aouba A, Deshayes S, Repesse Y (2023). Drug-associated acquired hemophilia A: an analysis based on 185 cases from the WHO pharmacovigilance database. Haemophilia.

